# AT1 Receptor Gene Polymorphisms in relation to Postprandial Lipemia

**DOI:** 10.1155/2012/271030

**Published:** 2011-09-19

**Authors:** B. Klop, T. M. van den Berg, A. P. Rietveld, J. Chaves, J. T. Real, J. F. Ascaso, R. Carmena, J. W. F. Elte, Manuel Castro Cabezas

**Affiliations:** ^1^Department of Internal Medicine, Center for Diabetes and Vascular Medicine, St. Franciscus Gasthuis, P.O. Box 10900, 3004 BA Rotterdam, The Netherlands; ^2^Department of Endocrinology, University of Valencia, 46010 Valencia, Spain; ^3^Unidad de Genotipado y Diagnóstico Genético, Fundación Investigación Clínico de Valencia-INCLIVA, 46010 Valencia, Spain

## Abstract

*Background*. Recent data suggest that the renin-angiotensin system may be involved in triglyceride (TG) metabolism. We explored the effect of the common A1166C and C573T polymorphisms of the angiotensin II type 1 receptor (AT1R) gene on postprandial lipemia. *Methods*. Eighty-two subjects measured daytime capillary TG, and postprandial lipemia was estimated as incremental area under the TG curve. The C573T and A1166C polymorphisms of the AT1R gene were determined. *Results*. Postprandial lipemia was significantly higher in homozygous carriers of the 1166-C allele (9.39 ± 8.36 mM*h/L) compared to homozygous carriers of the 1166-A allele (2.02 ± 6.20 mM*h/L) (*P* < 0.05). Postprandial lipemia was similar for the different C573T polymorphisms. *Conclusion*. The 1166-C allele of the AT1R gene seems to be associated with increased postprandial lipemia. These data confirm the earlier described relationships between the renin-angiotensin axis and triglyceride metabolism.

## 1. Introduction

Hypertriglyceridemia is an independent risk factor for the development of cardiovascular disease (CAD) [1, 2]. Recent prospective studies have shown that nonfasting TG levels are also associated with an increased risk in cardiovascular disease [3] and are possibly an even stronger predictor for CAD than fasting TG considering that humans are mostly in the nonfasting state [4, 5]. There is increasing interest to identify genes involved in the regulation of postprandial lipemia. The classical genes influencing TG metabolism like lipoprotein lipase or the APOE receptor are well known, but several others have been identified recently [6–8]. The large individual variability of TG levels cannot be fully explained by effects of these genes only. Most likely, environmental and dietary effects are the most important determinants of TG levels. However, unexpected effects by non-lipid-related genes have also been described. The renin angiotensin system mutational polymorphisms has been related to the metabolic syndrome and consequently to hypertriglyceridemia [9]. In the last decade, the beneficial effect of blockade of the renin-angiotensin system have been demonstrated in a wide variety of cardiovascular diseases, from heart failure to stable coronary artery disease and diabetic as well as nondiabetic chronic nephropathies [10, 11]. Therefore, the aim of the present study was to explore possible relationships between the angiotensin II type 1 receptor (AT1R) polymorphisms, A1166C and C573T, and fasting and postprandial triglyceridemia.

## 2. Material and Methods

### 2.1. Subjects

This paper is part of an ongoing study aimed at evaluating inheritable risk factors for premature atherosclerosis, metabolic disturbances, and genetic determinants. Normocholesterolemic CAD patients and their first-degree relatives were asked to participate. All CAD index patients had coronary sclerosis established by coronary angiography at a young age (before the age of 50 in men and of 60 in women) and had undergone a percutaneous transluminal coronary angioplasty (PTCA) at the Heart Lung Centre Utrecht. Exclusion criteria for index patients were the presence of diabetes mellitus, body mass index (BMI) >30 kg/m^2^, renal and/or liver failure, fasting plasma cholesterol >6.5 mmol/L (without lipid lowering medication), the presence of the apo E2/E2 genotype, the use of alcohol of more than 3 units a day, and a cardiac event or revascularization procedure during six months before the start of the study. Lipid-lowering medication, which was used by five patients, was stopped for 5 weeks before entering the study.

Only families of which at least two first-degree relatives were available for analysis were included. Exclusion criteria for family members were similar to those for the CAD index patients, except they should not have a medical history of CAD. Information about subjects' personal and family histories of cardiovascular disease was obtained by a standardised questionnaire. All participants were invited for a screening visit at the hospital. On the morning of inclusion blood pressure, weight, length, and waist and hip circumference were measured and a fasting blood sample was obtained for baseline determinations. The study protocol was approved by the Human Investigation Review Committees of the University Medical Centre Utrecht, and written informed consent was obtained from all participants.

### 2.2. TG Self-Measurements

Self-measurement of capillary TG was performed with a TG-specific point-of-care testing device (Accutrend GCT, Roche Diagnostics, Germany) [12–14]. Subjects were instructed to wash and dry their hands thoroughly before each measurement. With a lancing device, a drop of blood (30 *μ*L) from the finger was obtained which was applied to the TG test strip in the TG analyser. Subsequently, TG concentrations were measured by a process of dry chemistry and colorimetry. Participants measured their capillary TG on three different days at six standardized time points: fasting, before and three hours after lunch and dinner, and at bedtime. The results were recorded in a diary. Subjects were requested to refrain from heavy physical activity on the measurement days. Participants did not receive recommendations concerning the frequency and composition of the meals and were requested to use their regular diet during the study. In case of one or more missing measurements during a day, the data for that particular day were not used for construction of an average diurnal TG profile. The mean diurnal TG profile of 2 or 3 days was used for statistical analysis.

The measurement range of the Accutrend GCT for capillary TG is 0.80 to 6.86 mmol/L. The Accutrend system detects TG reliably, regardless of the nature of the triglyceride-carrying lipoprotein species (chylomicrons or VLDL particles) [15]. Variation coefficients for different capillary TG concentrations are 3.3% for high TG (6.12 mmol/L) and 5.3% for low TG (1.81 mmol/L) [15]. The correlation coefficient between Accutrend capillary TG measurements and plasma measurements according to enzymatic methods is 0.94 [15]. Furthermore, in healthy lean subjects diurnal capillary TG profiles correlate to postprandial lipemia assessed by standardised oral fat-loading tests [13]. In addition, diurnal triglyceridemia estimated with 6 measurements over the day was not different compared to hourly measurements, suggesting that the chosen time points are representative for the daylong study period [14].

### 2.3. Analytical Methods

Blood was collected at inclusion after a 12 hours fast for measurement of plasma lipids, apolipoproteins, insulin, and glucose. Cholesterol, plasma triglyceride, and HDL cholesterol (obtained after precipitation with dextran sulphate/MgCl_2_) were determined using a Vitros 250 analyser (Johnson & Johnson Rochester, NY, USA). Plasma apo B was measured by nephelometry using apo B monoclonal antibodies (Behring Diagnostics NV, OSAN 14/15). Plasma apo AI was measured by nephelometry using apo AI monoclonal antibodies (Behring Diagnostics NV, OUED 14/15). Plasma glucose was measured by glucose oxidase dry chemistry (Vitros GLU slides) and colorimetry and insulin was measured by competitive radio immunoassay with polyclonal antibodies. The HOMA-IR index (= glucose (mmol/L) *insulin (mU/L)/22.5) was calculated for estimation of insulin sensitivity.

### 2.4. Genotyping Procedures

Genotypes were determined as described previously [16]. A blood sample for polymorphism analysis was obtained from each patient in the morning after a minimum of 8 h fasting. Genomic DNA was extracted from white blood cells using silica gel polymer [17]. Reactions were conducted using DNA amplification in a final volume of 15 *μ*L containing 0.75 *μ*mol/L of each primer, 75 *μ*mol/L of each NTP, 2 ng/*μ*L DNA, 1.5 mmol/L MgCl_2_, 75 mmol/L Tris-HCl (pH 9.0), 20 mmol/L (NH_4_)_2_SO_4_, 5 mmol/L KCl, and 0.2 U/*μ*L Netzyme DNA polymerase (Need, SL, Valencia, Spain). The DNA was amplified for 40 cycles with denaturation at 94°C for 90 s (PTC-100 thermal cycler, MI Research). The polymerase chain reaction (PCR) products underwent electrophoresis using 2% agarose gel. DNA was visualized with ethidium bromide staining. The region of the *AT1R* located between nucleotides 423 and 1278 of the cDNA was amplified using oligonucleotides 5′-GGC TTT GCT TTG TCT TGT TG and 5′-AAT GCT TGT AGC CAA AGT CAC CT as sense and antisense primers, respectively. Amplification was conducted as described above. The A1166C and C573T polymorphisms of the *AT1R* gene were analyzed simultaneously by PCR using the technique which is described in detail elsewhere [18]. 

### 2.5. Statistical Analysis

Values are given as mean ± standard deviation (SD). Comparisons between CAD index patients and healthy relatives were performed with Student's *t*-test. Comparisons between the different genotypes of the polymorphisms were performed with one-way ANOVA with Bonferroni correction for parametric data and Pearson's Chi-square for nonparametric data. In the case of TG, insulin, and HOMA index, calculations were performed after logarithmic transformation; however, untransformed concentrations are shown in the text, tables, and figures. Calculations of TG-AUCs were performed with GraphPad Prism version 3.0 for Windows (GraphPad Software, San Diego, Calif, USA) using nonlogarithmic transformed TG concentrations. Postprandial lipemia was defined as the incremental area under the capillary TG curve after correction for fasting capillary TG (dTG-AUC). For statistical analysis, PASW version 18.0 was used. Statistical significance was reached when *P* < 0.05 (two sided).

## 3. Results

A total of 82 subjects were included. Sixteen of them were normocholesterolemic CAD patients, and there were 66 healthy relatives. The frequency of the T-allele for the C573T gene polymorphisms was 65%. The C-allele for the A1166C gene polymorphisms showed a frequency of 60%. No significant differences in allele distribution between patients and family members were found. All polymorphisms were in Hardy–Weinberg equilibrium. Baseline characteristics for the A1166C and C573T polymorphisms are shown in Tables 1 and 2, respectively. No significant differences were found among the different A1166C polymorphisms, but the BMI was significantly different among the C573T polymorphisms (*P* = 0.047). 

**Figure 1:**
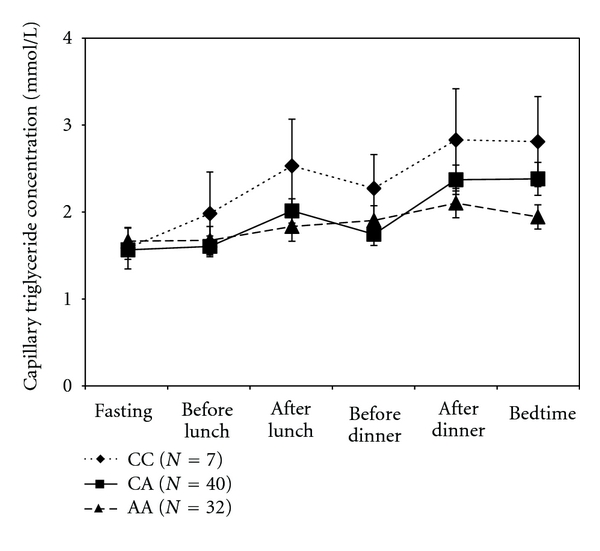
Diurnal capillary triglyceride (cTG) patterns for A1166C polymorphisms. Fasting cTG was similar for the three different polymorphisms, but homozygous carriers of the 1166-C allele showed a significantly increased incremental area under the capillary triglyceride curve compared to homozygous carriers of the 1166-A allele (*P* < 0.05).

**Figure 2:**
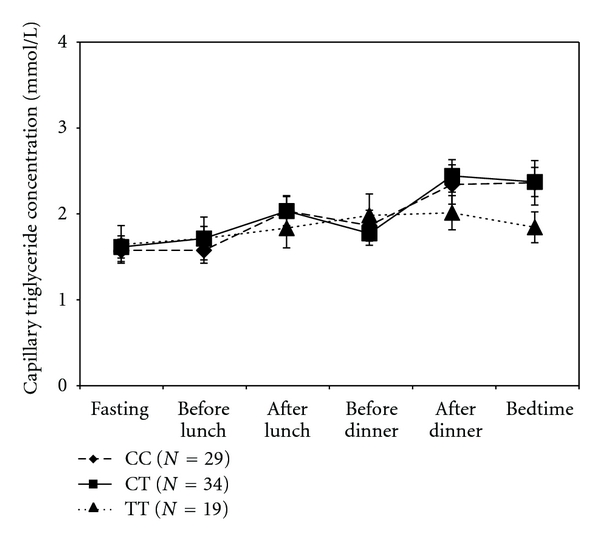
Diurnal capillary triglyceride patterns for C573T polymorphisms. No significant differences for the polymorphisms were found in the incremental area under the capillary triglyceride curve as a marker for postprandial lipemia.

Postprandial lipemia expressed as the dTG-AUC was significantly higher in homozygous carriers of the 1166-C allele (9.39 ± 8.36 mM*h/L) compared to homozygous carriers of the A-allele (2.02 ± 6.20 mM*h/L) (*P* < 0.05). Postprandial lipemia in carriers of the CA polymorphism of the A1166C gene was 3.65 ± 7.42 mM*h/L, which was intermediate to the CC and AA polymorphisms, without reaching statistical significance (Figure 1). Postprandial lipemia was similar for the different C573T gene polymorphisms ranging from 4.05 ± 9.54 mM*h/L for the CC polymorphism, 3.82 ± 4.75 mM*h/L for the CT polymorphism, to 2.17 ± 7.00 mM*h/L for the TT polymorphism (Figure 2).

## 4. Discussion

It has been suggested that 40% of the variation in triglyceride concentrations in the population is caused by genetic heritability [19]. This study shows for the first time increased postprandial lipemia in homozygous carriers of the 1166-C allele of the AT1R gene. It should be noted that the number of subjects in this study was small and only seven subjects were homozygous carriers of the 1166-C-allele. Despite the fact that fasting TG, the strongest determinant of postprandial lipemia, was similar for the groups, the CC-group showed an exaggerated postprandial response. Disturbances of lipid metabolism are frequent in patients with hypertension, metabolic syndrome, and cardiovascular disease; together they share numerous susceptibility genes [6]. Two experimental studies demonstrated increased contraction of human arteries in homozygous carriers of the 1166-C allele [20, 21]. In hypertensive patients with metabolic syndrome, the presence of the CC1166 genotype was a risk factor for central obesity and dyslipidemia [9]. Furthermore, the C-allele of the A1166C polymorphism has been associated with a decreased endothelial response to statin treatment measured with brachial artery flow-mediated dilation [22]. The C-allele also predisposes to an increased risk for stroke, especially in combination with hypertension [23]. It is known that endothelial function becomes impaired by increased TG concentrations after an oral fat-loading test [24, 25]. All these data suggest that the A1166C polymorphism of the AT1R gene shares susceptibility with common disorders seen in metabolic syndrome like postprandial lipemia.

The C573T polymorphism did not show any differences in postprandial lipemia. A possible explanation is that it does not influence the amino acid sequence of the encoded protein although this polymorphism is located in the coding region of the gene [21]. In contrast, the A1166C polymorphism is located in a nontranslated region of the AT1R gene but it has been suggested to be in linkage disequilibrium with another, yet-unknown, functional mutation thus explaining the multiple associations of this polymorphism with several diseases. However, contradictory results have also been found which could not confirm the association of the A1166C polymorphism with metabolic syndrome, hypertension, or cardiovascular disease [26, 27]. In one study the A1166C polymorphism was not related to triglyceride concentrations, but this study was performed in a Hong Kong Chinese population and only two subjects with the CC genotype could be identified [28]. A very recent study comprising more than 100,000 individuals did not find an association between the A1166C polymorphism and blood lipids, including TG [8]. However, the authors did not study specifically postprandial lipemia.

## 5. Conclusions

Although contribution of the different genes seems small, there is evidence that genetic variations have a cumulative effect on the cardiovascular risk [18, 29, 30] and that genetic variations modulate the effect of environmental factors on cardiovascular risk [31]. With the availability of easy and quick gene mapping, it might be possible in the future to genetically classify patients according to their risk, before the disease phenotype is manifest. This might lead to preventive treatment of patients in the high-risk classes, thus diminishing disease burden and preventing unnecessary treatment of low-risk patients. In conclusion, we report that postprandial lipemia is increased in homozygous carriers of the C-1166 polymorphism of the AT1R gene. No association was found between postprandial lipemia and the C573T polymorphism.

## Figures and Tables

**Table 1:** Baseline characteristics for A1166C polymorphisms. Data are given as mean ± standard deviation unless stated otherwise.

	AA (*N* = 34)	CA (*N* = 41)	CC (*N* = 7)	*P*-value
Age (years)	43.8 ± 16.5	41.3 ± 11.0	50.0 ± 12.1	NS
Male gender	18 (53%)	22 (54%)	3 (43%)	NS
CAD	9 (26%)	5 (12%)	2 (29%)	NS
BMI	25.1 ± 3.4	25.6 ± 4.7	26.5 ± 2.9	NS
Waist (cm)	91.8 ± 11.3	92.0 ± 15.6	96.0 ± 11.8	NS
Systolic RR (mmHg)	126.6 ± 10.6	124.2 ± 14.5	127.9 ± 10.4	NS
Diastolic RR (mmHg)	82.8 ± 9.2	82.1 ± 8.1	84.3 ± 6.1	NS
Total cholesterol (mmol/L)	5.5 ± 0.9	5.3 ± 1.0	5.6 ± 0.6	NS
LDL-C (mmol/L)	3.7 ± 0.8	3.5 ± 0.9	3.6 ± 0.7	NS
HDL-C (mmol/L)	1.26 ± 0.33	1.18 ± 0.30	1.35 ± 0.40	NS
Plasma TG (mmol/L)	1.34 ± 0.62	1.37 ± 0.70	1.29 ± 0.79	NS
ApoB (g/L)	0.99 ± 0.23	0.93 ± 0.23	0.94 ± 0.20	NS
ApoA-I (g/L)	1.37 ± 0.19	1.33 ± 0.23	1.30 ± 0.28	NS
HOMA-IR	2.31 ± 1.41	2.51 ± 1.96	2.70 ± 1.75	NS

CAD: coronary artery disease, BMI: body mass index, RR: blood pressure, TG: triglyceride, Apo: apolipoprotein, HOMA-IR: homeostatic model assessment.

**Table 2:** Baseline characteristics for C573T polymorphisms. Data are given as mean ± standard deviation unless stated otherwise.

	CC (*N* = 29)	CT (*N* = 34)	TT (*N* = 19)	*P*-value
Age (years)	45.0 ± 10.4	40.7 ± 15.4	44.3 ± 15.0	NS
Male gender	13 (44.8%)	20 (58.8%)	10 (52.6%)	NS
CAD	6 (20.7%)	4 (14.7%)	5 (26.3%)	NS
BMI	26.6 ± 5.0	25.5 ± 3.6	23.7 ± 2.5	*P* = 0.047
Waist (cm)	95.2 ± 15.7	92.0 ± 12.8	88.2 ± 10.5	NS
Systolic RR (mmHg)	125.6 ± 10.9	125.3 ± 14.9	125.8 ± 11.2	NS
Diastolic RR (mmHg)	84.0 ± 7.5	80.6 ± 8.0	84.2 ± 9.8	NS
Total cholesterol (mmol/L)	5.6 ± 1.0	5.2 ± 0.8	5.4 ± 1.0	NS
LDL-C (mmol/L)	3.8 ± 1.0	3.4 ± 0.8	3.5 ± 0.9	NS
HDL-C (mmol/L)	1.29 ± 0.34	1.17 ± 0.29	1.24 ± 0.34	NS
Plasma TG (mmol/L)	1.27 ± 0.55	1.43 ± 0.73	1.33 ± 0.73	NS
ApoB (g/L)	0.97 ± 0.22	0.95 ± 0.22	0.94 ± 0.26	NS
ApoA-I (g/L)	1.35 ± 0.21	1.34 ± 0.23	1.35 ± 0.20	NS
HOMA-IR	2.77 ± 2.34	2.45 ± 1.40	2.45 ± 1.72	NS

CAD: coronary artery disease, BMI: body mass index, RR: blood pressure, TG: triglyceride, Apo: apolipoprotein, HOMA-IR: homeostatic model assessment.
